# Effect of organic solvent additives on the enhancement of ultrasonic cavitation effects in water for lithium-ion battery electrode delamination

**DOI:** 10.1016/j.ultsonch.2024.107049

**Published:** 2024-08-31

**Authors:** Chunhong Lei, Ben Jacobson, Jennifer M. Hartley, Sean Scott, Iwan Sumarlan, Andrew Feeney, Paul Prentice, Karl S. Ryder, Andrew P. Abbott

**Affiliations:** a School of Chemistry, University of Leicester, Leicester LE1 7RH, UK; b James Watt School of Engineering, University of Glasgow, Glasgow G12 8QQ, UK; c Department of Chemistry, University of Mataram, Lombok, Indonesia

**Keywords:** Ultrasonic delamination, Cavitation strength, Lithium-ion battery, Solvent additive, Cavitation detector, Sonochemiluminescence

## Abstract

Ultrasonic delamination is a low energy approach for direct recycling of spent lithium-ion batteries. The efficiency of the ultrasonic delamination relies both on the thermophysical properties (such as viscosity, surface tension, and vapour pressure) of the solvent in which the delamination process is carried out, and the properties of the ultrasound source as well as the geometry of the containment vessel. However, the effect of tailoring solutions to optimise cavitation and delamination of battery cathode coatings has not yet been sufficiently investigated. Acoustic detection, high-speed imaging, and sonochemiluminescence (SCL) are employed to study the cavitation processes in water-glycol systems and identify the effect of tailoring solvent composition on cavitation strength. The addition of small volume fractions of organic solvent (ca. 10–30 vol%), including ethylene glycol or glycerol, to the aqueous delamination solution were found to significantly improve the delamination efficiency of lithium-ion battery cathode coatings due to the alteration of these thermophysical properties. However, greater volume fractions of glycol decrease delamination efficiency due to the signal-dampening effect of viscosity on the ultrasonic waves. The findings of this study offer valuable insights for optimising ultrasonic bath solution composition to enhance film delamination processes.

## 1 Introduction

With the rapid increase in retired lithium-ion batteries (LiBs) from electric vehicles, the recycling of spent LiBs has become increasingly urgent to ensure the health and sustainability of the industry [1]. Several national governments have set zero emissions mandates, which has contributed to an increase in EV sales [2]. Legislation is also being adopted that requires a minimum quantity of recycled materials in new EV batteries. For example, the EU will require that a minimum amount of the lithium, nickel and cobalt used in LiB cathode materials (10 %, 12 % and 15 % respectively) will have to be sourced from recycled materials by 2035 [3]. Current recycling methodologies often result in these valuable components being recovered in the form of precursors, while the less easily recycled materials such as graphite, electrolyte, or plastics are lost [4], [5]. One current topic of research is the concept of ‘direct recycling’, where the aim is to retain the original structure and performance of the battery active materials, either through relithiation or direct reuse [6], [7], [8], [9]. However, the effectiveness of direct recycling approaches often depends upon prior separation processes, to ensure no cross-contamination of the waste streams as this can impact recycling efficiency or the performance of the second-life batteries [10].

Ultrasonic delamination has recently been investigated for separating cathode and anode active material coatings from the metal foil current collectors, where ultrasound has the potential to significantly increase reaction rates, reducing the time frames required from hours and minutes to minutes and seconds [4]. High-intensity ultrasound is already demonstrated as an effective technique for processing of material surfaces, such as printed circuit boards [11], [12] and free-floating intermetallics [13]. Primarily, the cavitation generated by sonication at sufficient intensities to cause cavitational collapse is the major driver of several phenomena that result in high velocity fluid circulation, which is important for surface treatment [14]. These phenomena include the generation of high-pressure bubble collapse shockwaves, powerful microjets impacting the material surface, and enhanced fluid circulation at the material surface resulting from bubble cluster microstreaming and acoustic streaming from the ultrasound source [11], [13], [15]. Typically, water-based solutions are preferred for their low flammability, eco-friendliness, and affordability in comparison to organic solvents. However, additives have been incorporated to modify the solution properties, such as surface tension, density, viscosity, and vapour pressure, in order to enhance cavitation effectiveness. Surfactants such as sodium dodecyl sulphate have been shown to alter cavitation patterns by modifying solution properties such as viscosity and surface tension [16]. Organic additives have previously been shown to increase the cavity energy in aqueous solutions. For example, Khavari et al. reported an increase in cavitation activity upon the addition of ethanol to water, [17] whereas Wang et al. reported the attenuation rate of ultrasonic and cavitation energy in aqueous solution with glycerol and ethanol, showing that the ultrasonic energy attenuation is a combined result of solution physical properties, the number, and size of cavitation bubbles [18].

For LiB anode and cathode delamination, both high- and low-power (above and below 10 W/cm^2^) methods have been used, with a range of solvents, including organic solvents such as NMP, or aqueous solutions containing alkalis such as NaOH, organic acids such as oxalic or citric acid, or neutral additives such as ethylene glycol [19], [20], [21], [22], [23], [24]. The LiB cathode was made with valuable lithium nickel manganese cobalt oxide (LiNiMnCoO_2_) compound particles, coated on thin aluminium foil current collectors (ca. 15 μm thick), with the aid of a polyvinylidene fluoride (PVdF) binder. It is an important recycling step to separate the LiNiMnCoO_2_ coating from the aluminium foil without damaging the aluminium foil. We have previously demonstrated the fast ultrasonic delamination of LiB cathodes, with a sample movement rate of 2 cm/s under a sonotrode blade [19]. High delamination strength and therefore the choice of a solution is crucial for fast ultrasonic delamination. The choice of solvent and ultrasonic power was strongly dependent on the type and amount of binder present, electrode chemistry, such as whether graphite, LFP or NMC is present, and which metal ion ratios are used, and hence the composition of cathode electrolyte interphase formed during charging-discharging. The different properties of these electrodes will impact which interface is exploited to cause delamination and the amount of voids present, which facilitates the process. High-intensity ultrasound (ranging from 10 to 1,000 W/cm^2^ of the emitting surface) or high wave pressure is essential for rapid delamination of both anodes and cathodes [25], with the latter being particularly challenging to delaminate due to the smaller particle sizes and increased binder content resulting in fewer voids within the coating.

The aim of this work is to characterise the effects of ethylene glycol and glycerol content on the cavitation behaviour of aqueous glycol solutions under the application of high-power ultrasound, as a means to optimise the delamination efficiency of LiB cathode coatings. These additives will alter the viscosity, density, surface tension, and vapour pressure of the solutions. Aqueous solutions of ethylene glycol and glycerol were selected as delamination solutions because both glycols can act as wetting agents towards PVdF and will alter the aforementioned physical properties of the solution, without affecting the solution pH, or degrading either the cathode active materials or the aluminium foil current collectors. These additives have low toxicity and low flammability. A high-speed camera is used in combination with a shockwave passive cavitation detector (swPCD) to characterise the cavitation activity in the solution, revealing the connection between cavitation behaviour and solution properties.

## 2 Experimental methods

### 2.1 Chemicals and solutions

Deionised water (18.2 Ω, Elga Purelab Option apparatus), ethylene glycol (EG, Sigma, 99.8 %), and glycerol (Gly, Fisher, 99 %) were used without further purification. Delamination solutions were made by mixing EG or Gly with deionised water in ratios of 10 to 50 v/v% and stirring at room temperature until a homogenous solution had been obtained.

### 2.2 The experimental setup

**Fig. 1:**
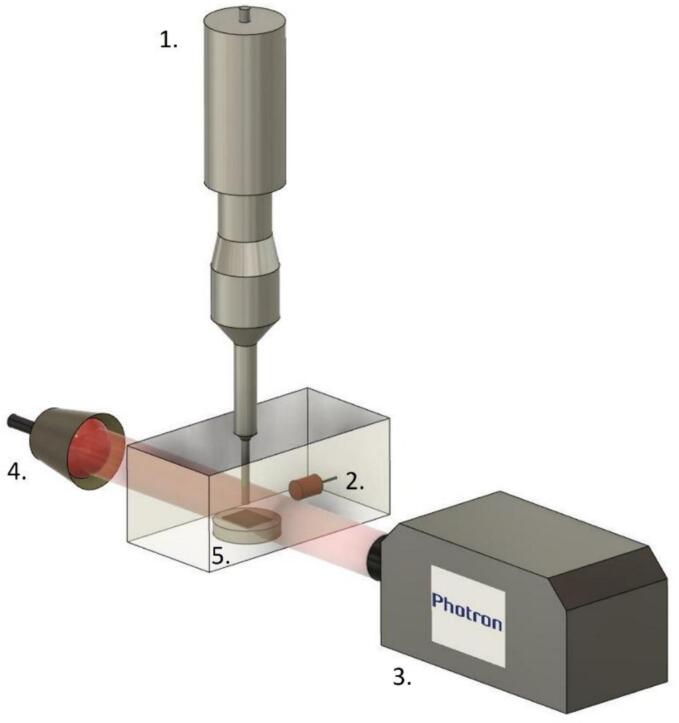
Schematic representation of the experimental setup, featuring the following components: 1. The sonotrode, submerged in the solution within a custom-made tank. 2. The swPCD detecting acoustic cavitation emissions. Photron high-speed camera. 3, imaging cavitation at the vicinity of the sonotrode tip with illumination provided by pulsed laser. 4. For delamination and erosion experiments, LiB sheets were mounted below the sonotrode, denoted by 5.

The characterisation of cavitation activity in each solution was carried out with the same equipment as the LiB delamination experiments, with one exception: the lack of a LiB sheet in the former experiment. Results presented were obtained using two different transducers immersed in a Perspex tank, represented by component 1, Fig. 1. Firstly, a sonotrode with a diameter of 6 mm (Ultrasonic Processor VC-505) with a variable power of up to 500 Watts. Secondly, a sonotrode with a diameter of 20 mm (Branson Sonics, 1.25DCXa20-V) with a variable power of up to 1250 Watts, both operating at 20 kHz. All experiments were conducted at room temperature (ca. 20 °C±3 °C).

### 2.3 Acoustic detection and high-speed imaging

Acoustic detection of cavitation emissions was undertaken with a bespoke in-house fabricated swPCD (component 2, Fig. 1) based on 110 μm thick PVdF and designed for high-sensitivity to bubble-collapse shockwaves [26]. The swPCD used in this study incorporates an active element that is 10 mm in diameter, which was mounted on a 3-D positioning system within the tank for the detection of emissions orthogonally with respect to the sonotrode tip.

Cavitation emissions, detected by the swPCD (in millivolts, mV) were collected by an oscilloscope (Tektronix 5 series, Berkshire UK) at a sample rate of 25 × 10^6^ samples/s. A filtering protocol was applied to reduce noise (low-pass < 10 MHz) and f_0_ (high-pass > 20 kHz), revealing shockwave content for presentation in the voltage–time domain, quantified by the root mean square (V_RMS_) of the emission signal averaged over five 200 ms duration samples per power. A high-speed camera (Fastcam SA-Z 2100 K, Photron, UK) (component 3*,* Fig. 1) is used to monitor the dynamics of the cavitation in the water-glycol solutions as well as directly image the delamination of the LiB samples at 5000 to 80,000 frames per second (fps). The illumination was provided via synchronous 10 ns laser pulses at 640 nm (CAVILUX Smart, Cavitar, Finland) (component 4*,* Fig. 1).

The contact angle measurement of the different solvents on a PVdF film was carried out in air at room temperature, with a contact angle machine (ThetaLite101, Biolin Scientific). The liquid droplet was delivered manually through a flat-edge syringe. The droplet volume was about 30 µL, the contact angle measurement of each droplet was taken after 5 s in contact with the PVdF film.

### 2.4 Delamination and erosion

The delamination of LiB cathode was carried out in deionised water, and with different vol% of EG or Gly, using the Branson transducer operating through the 20 mm-Ø sonotrode at an intensity of 119 W/cm^2^ (30 % power output). Samples of LiB cathodes for delamination were cut into 3 cm × 3 cm squares from a larger sheet and mounted onto a stainless-steel cylinder, with a 5 mm separation distance under the sonotrode tip (component 5*,* Fig. 1). The cathode was made from an aluminium foil current collector (15 μm thick), coated on each side with 80 μm of LiNiMnCoO_2_, with ca. 7 wt% of PVdF binder. The cathode sheet was uncycled and unwetted with electrolyte. Samples were sonicated for 10 s, and the delamination rate was evaluated by photographing. This process was repeated for 5 samples for each solution to mitigate any effects due to the location of the cathode sheet from which the samples were taken.

The erosion of aluminium foils under ultrasonic conditions was tested on two types of aluminium sheet: thick aluminium plate (0.5 mm thick, Rapid Electronics Ltd, 99.5 %), and thin aluminium foil (30 μm thick, Korff, VWR International). Samples were mounted onto a stainless-steel holder using Kapton tape. Ultrasound was applied using the same Branson sonotrode at an intensity of 119 W/cm^2^. A sample sheet was positioned on a stainless-steel cylinder at 2.5, 5.0, or 10.0 mm beneath the sonotrode tip, immersed in deionised water, and exposed to sonication for 3 s.

### 2.5 Sonochemiluminescence (SCL)

To visualise the active cavitation regions surrounding the sonotrode tip, monitoring of the sonochemiluminescence (SCL) during a sonication was performed. This involves sonicating of aqueous solution of luminol (5-amino-2,3, dihydro-1,4-phthalazinedione) under alkaline conditions. Luminol reacts with the sonochemically produced hydroxyl (OH^•^) radicals and the final product of the reaction (3-amino phthalate) emits blue light [27]. Effectively, when cavitation bubbles implode, they create high-pressure and high-temperature regions, triggering the formation of OH^•^ radicals. The intensity of SCL is directly proportional to the density of cavitation activity. Therefore, higher illumination intensity indicates a higher density of bubbles and more violent cavitation.

For the monitoring of SCL, 20 mL solutions of 5.0 M sodium hydroxide (NaOH) (98 %, Fisher Scientific) were diluted with 980 mL of water or equivalent volume of water/organic solvent volume fraction ratio. To this, 0.17 g of luminol was added (>97 %, Sigma Aldrich). This ratio provided the brightest SCL emission. Imaging was captured in a light insulated box within a dark room using a Nikon D5600 camera and NIKKOR lens (AF-S 50 mm 1:1.8 G). The exposure time was 10 s, aperture f/2 and ISO setting was 1600.

## 3 Results and Discussion

The results below are organised as follows: 3.1, 3.2, 3.3 present the characterisation of cavitation behaviour in the various water-glycol blends. Cavitation behaviour is characterised acoustically through the time-averaged shockwave content (*V_RMS_*) in each liquid at various glycol content with additional characterisation through high-speed imaging and SCL. 3.4, 3.5 present delamination experiments on LiB samples for assessment of cavitation effectiveness at each water-glycol blend composition with further investigation into the erosive force of the cavitation.

### 3.1 Acoustic detection

**Fig. 2:**
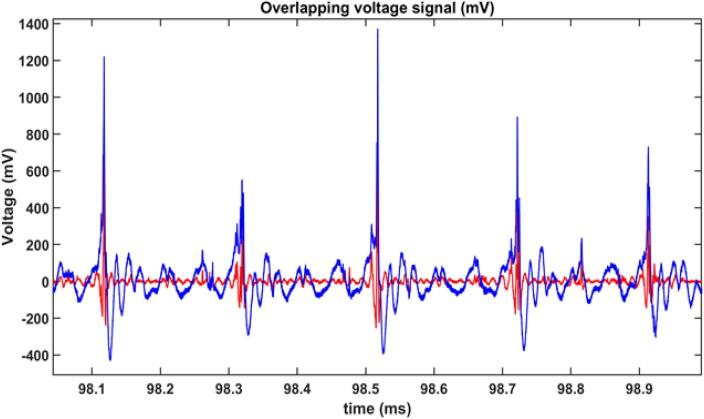
Example of signals from the swPCD located beside a 6 mm-Φ sonotrode in a solution of deionised water with 10 v/v% EG. Blue line: raw signal from swPCD. Red line: filtered raw signal showing shockwave signal from cavitation.

**Fig. 3:**
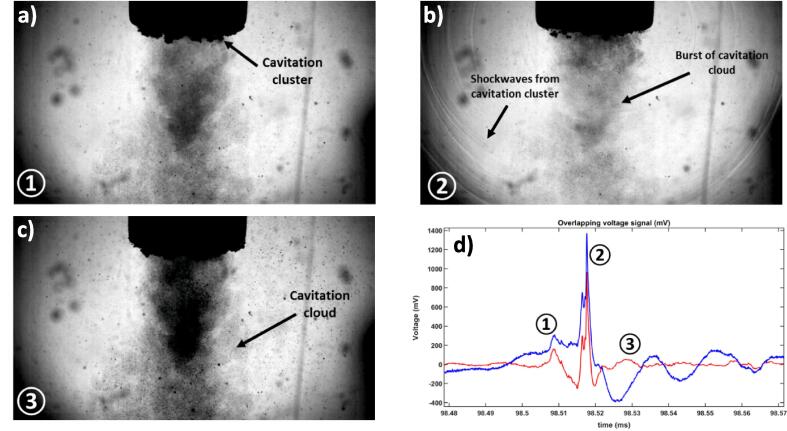
Consecutive high speed camera images (80 kfps, 0.0125 ms interval), a), b), c), and corresponding swPCD signal, d), at position ①, ② and③, with the 6 mm-Φ in a solution of deionised water with 10 v/v% EG.

Fig. 2 illustrates a typical swPCD signal recorded during a sonication from the 6 mm-Φ sonotrode operating at a power density of 354 W/cm^2^ (20 % power output) in a solution of deionised water with 10 v/v% EG over a period of ca. 1 ms. The swPCD was positioned 2 cm alongside the sonotrode tip. The blue line represents the raw signal, while the red line depicts the filtered signal, highlighting the bubble collapse shockwaves. The acoustic signal detected by the swPCD gives a quantitative measure of the amplitude of shockwaves generated by the cavitation bubble clouds surrounding the sonotrode tip as well as an allowing for observation of the periodicity (number of acoustic cycles between shockwaves) of the bubble collapse shockwaves. As observed in Fig. 2, higher amplitude peaks are seen to occur every 3 to 4 cycles of the sonotrode driving frequency, between 400 and 1,000 mV higher than the fundamental acoustic waves. This observation has been demonstrated and explained in detail in water under a sonotrode [28]. These major peaks coincide with the intense periodic cavitational collapse of the primary cone-like bubble cluster that is formed at the distal end of the sonotrode [29], [30]. This is better realised in the high-speed imaging snapshot images in Fig. 3.

Fig. 3 depicts representative frames from a high-speed imaging sequence of the cavitation under the sonotrode, captured at 80,000 fps, corresponding to a period of 0.0125 ms and synchronised with the acoustic detection of the swPCD. The first image (Fig. 3**a**) shows the area under the sonotrode as the cavitation cluster is forming, immediately before the bubble cloud collapses. The formation of the bubble cloud under the sonotrode tip is often described as cone-like, which is typically formed due to pressure differentials at the tip resulting in bubble translation and coalescence [29]. The extent of the bubble cloud formation is determined by the diameter of the sonotrode tip as well as the intensity of the source. Generally, the behaviour of the bubble cluster under the sonotrode tip is synchronised, meaning the bubbles oscillate and collapse over the same number of cycles. Illustrated in Fig. 3**b** is intense and multi-fronted shockwaves from collapses of the cavitation cluster, corresponding to the sharp peaks at the position ② of the swPCD signal, originating from throughout the large bubble cluster, propagating throughout the medium. Alongside the growth, oscillation and violent collapse of the bubble cloud under the sonotrode, acoustic streaming is prevalent in the system. Fig. 3**c** depicts a tremendous bubble cloud growth and propagation down away from the sonotrode tip. Following the intense acoustic pressure oscillation generated by the shockwaves as depicted in position ③ in Fig. 3**d**. The large flows of finer bubbles were seen to be circulating down and away from the sonotrode tip. Here, the contribution of these bubbles on the shockwaves presented in the previous image is minor. However, acoustic streaming is an important phenomenon for enhancing vortex-like flow and increased mass transport at the interface of the solid surface.

### 3.2 Mechanism of acoustic pressure

The properties of the solution play crucial roles in determining the characteristics of cavitation, such as bubble formation, growth, collapse, and the resulting effects on targeted materials. Liquid density affects the buoyancy and stability of cavitation bubbles, vapour pressure influences bubble nucleation, surface tension affects bubble surface stability and dynamics, and viscosity determines the resistance to bubble expansion and collapse [31]. Understanding how these properties influence performance is vital for controlling and optimising cavitation processes in various applications such as homogenization, dispersion, erosion, emulsification, and sonochemistry. The dynamics of a spherical cavitation bubble with time-varying radius *R(t)* under an acoustic pressure can be described by the Rayleigh–Plesset equation (**Eq.1**) [32]:

(1) $$R\ddot{R}+\frac{3}{2}\dot{R^{2}}=\frac{p_{b}-p_{\infty }\left(t\right)}{\rho_{l}}+\frac{4v_{l}}{R}\dot{R}-\frac{2\sigma }{\rho_{l}R}$$

where: *R(t)* is the time-varying radius of the bubble, *p_b_(t)* is the pressure within the bubble, mainly the vapour pressure of the solution, *p*_∞_(t) is the external pressure infinitely far away from the bubble, *ρ_l_* is the density of the surrounding liquid, *ν_l_* is the kinematic viscosity of the surrounding liquid, and *σ* is the surface tension of the vapour-liquid interface. *p_∞_* (t) is the driving force for the formation and growth of cavitation bubbles, a higher *p_∞_* (t) value in a specific position leads to the generation of more cavitation bubbles in those local positions. Vapour pressure, which is in a positive direction, promotes bubble expansion and resists bubble shrinkage. As a result, it reduces the strength of cavitation implosions, making them less damaging to materials. Both EG and Gly have lower vapour pressures compared to water (Table S1) and will hence reduce the overall vapour pressure of the solution, with Gly reducing it more significantly than EG.

The viscosity of the solution plays a damping role in the expansion or contraction of cavitation bubbles. It reduces the amplitude of bubble oscillations and can also delay or limit the spreading range of acoustic waves and shockwaves. In solutions with higher viscosity, more vibration energy is converted into thermal energy due to increased friction. This conversion process further attenuates the propagation of acoustic waves and reduces their damaging effects. Both EG and Gly will increase the viscosity of the solution, with Gly causing a more significant increase compared to EG (Table S1). These combined effects influence the transmission of acoustic pressure and contribute to the observed trends in average shockwave content of the cavitation emissions (measured by *V_RMS_*) for EG-water and Gly-water solutions.

Variation in the surface tension of a solution can have implications on the amplitude of shockwave emissions, contributing to shockwave content throughout sonication, hence intensity of cavitation collapse. High surface tension in a solution can hinder the formation and growth of cavitation bubbles. When cavitation occurs in a solution with high surface tension, more elastic energy is stored in the bubble, leading to a stronger and more violent implosion upon collapse, increasing the *V_RMS_*. Conversely, low surface tension promotes the instability of bubble growth and collapse. This instability can cause the bubble to break up into smaller bubbles, reducing the destructive power. In essence, surface tension serves as a critical factor in determining the dynamics and effects of cavitation in a solution. The strength of the disturbances caused by cavitation is therefore closely linked to the properties of the solution in which the bubble is generated. Generally, cavitation with higher surface tension will be stronger than cavitation with lower surface tension. Therefore, the differences observed in surface tension between water-EG and water-Gly blends can influence the strength of cavitation in these solutions.

The surface tension of these aqueous solutions containing different vol% EG or Gly can be inferred by measuring the contact angle of the solution on a particular substrate, due to the following relationship (Young’s equation) **Eq. 2** [33]:

(2) $$\gamma_{\mathrm{LG}}=\frac{\gamma_{\mathrm{SG}}-\gamma_{\mathrm{SL}}}{{cos\theta }_{C}}$$

where: *γ_LG_* is the liquid–vapour interfacial energy (surface tension), *γ_SG_* is the solid-vapour interfacial energy, *γ_SL_* is the solid–liquid interfacial energy, and *θ_C_* is the contact angle. A smaller contact angle therefore corresponds to a smaller surface tension.

**Fig. 4:**
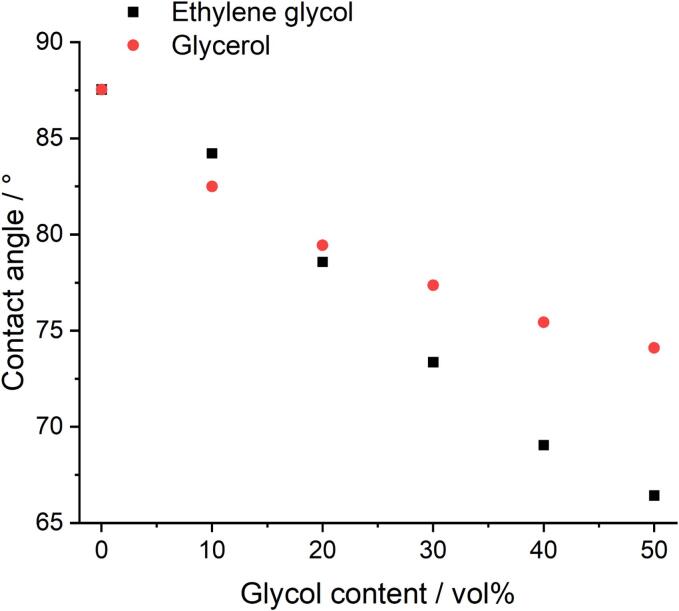
Contact angles of deionised water with different vol% of EG or Gly on a PVdF film coated onto a glass microscope slide, measured in air at room temperature. The original contact angle images can be found in Figures S2 & S3.

In this work, a PVdF film is used as the substrate, as this is the binder material most commonly found in LiB cathodes. While it would be more ideal to use the cathode sheet itself, the active material is porous and does not lend itself well to accurate contact angle measurements. Fig. 4 shows the decrease in contact angle of the solution on the PVdF film as a function of glycol content. These values are similar for both water-glycol blends, up to a glycol content of 30 vol%. Above a 30 vol% glycol content, the contact angles of the water-EG solutions decrease more rapidly with increasing EG content, in comparison to the water-Gly systems. This implies that the surface tension of each water-Gly solution is generally higher than the equivalent water-EG systems at glycol contents of greater than 30 vol%, and therefore should result in more violent bubble collapses. Literature values for surface tension support this observation. For example, at ca. 40 wt% glycol content, the surface tension for an EG solution is 52 mN/m, whereas for a Gly solution it is 70 mN/m [34], [35]. In general, the surface tension of the solution decreases with increasing glycol content.

**Fig. 5:**
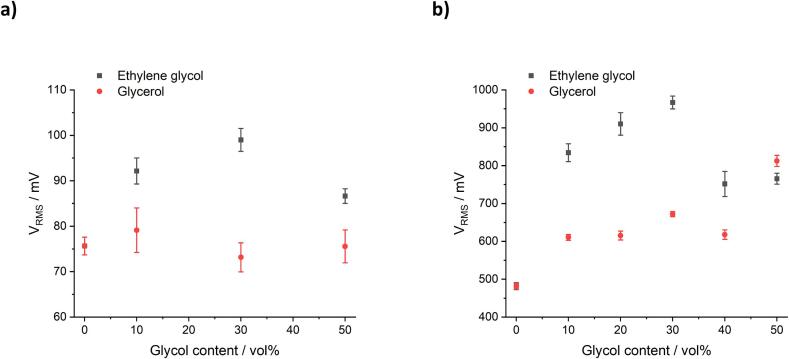
Root mean square of the voltage (*V_RMS_*) values obtained using a) a 6 mm-Φ sonotrode tip operating at a power density of 354 W/cm^2^, measured by a swPCD located 2 cm orthogonal to the sonotrode tip, and b) a 20 mm-Φ horn operating at a power density of 119 W/cm^2^, measured by a swPCD located 2 cm directly beneath the sonotrode. The measurements were conducted in deionised water with different vol% of EG or Gly. Note that the absolute magnitudes of *V_RMS_* are different due to the different transducers used. Results are presented as mean +/− standard deviation for five measurements.

To analyse the effect of solution properties on bubble collapse shockwave intensity, the Sonics transducer operating through the 6 mm-Φ sonotrode tip operating at a power density of 354 W/cm^2^, was employed. The sonotrode tip was immersed in a series of aqueous solutions containing EG or Gly, and the resulting acoustic emissions were detected by the swPCD. The time-averaged shockwave content was quantified using the root mean square of the voltage (*V_RMS_*) over five 200 ms duration samples per power, as per Yusuf et al. [28]. The results are presented in Fig. 5. The raw swPCD signal consists of all the pressure waves, including the soundwave of the driving frequency emitted from the Sonics transducer, the shockwaves from the cavitation cluster formed near the sonotrode tip, shocks from the collapse of individual or groups of cavitation bubbles on or near the swPCD surface, pulsation of the cavitation bubbles as they expand and collapse, and the reflected wave from the vessel walls. The signal from the swPCD can be expressed as **Eq.** (3) [25]:

(3) $$I={\left(\frac{p^{2}}{\rho c}\right)}_{\mathrm{average}}$$

where *I* is the intensity of sound waves, corresponding the strength of the ultrasound; *ρ* is the density of the solution; *c* is the speed of sound in the solution, *p* is peak pressure, and so (*p^2^*)_average_ is the mean square pressure, corresponding to the root mean square voltage *V_RMS_* obtained by the oscilloscope.

For the EG-water solution, the *V_RMS_* value initially increases with EG content, peaking at 30 % EG, before decreasing with further increases in EG content (Fig. 5**a**). This initial increase is likely due to the lower surface tension and increased viscosity with EG content. As the EG content increases further, the signal-dampening effect of viscosity decreases the *V_RMS_* value. On the other hand, for the Gly-water solution, the *V_RMS_* value remains relatively similar to that for deionised water as the Gly content increases. This indicates that the transfer of acoustic pressure away from the sonotrode axial line is greatly attenuated due to the higher viscosity of Gly solution.

For the Branson transducer with the 20 mm-Ø sonotrode and the swPCD placed directly under the sonotrode, the *V_RMS_* values for EG solutions follow the same trend as for when the swPCD is placed orthogonally to the sonotrode (Fig. 5**b**). In contrast, Gly concentration increases the *V_RMS_* value when the swPCD is directly under the sonotrode, indicating that the acoustic pressure emitted from the sonotrode in the Gly solution is more focused along the narrow column along the sonotrode axis line. This focused pressure distribution is attributed to the higher viscosity of Gly-water solution. As the concentration of additives increases, the *V_RMS_* value in water-Gly solution gradually increases to match or even surpass the *V_RMS_* value in the water-EG solution. This phenomenon could be attributed to the increased surface tension of water-Gly solution and so an enhanced cavitation activity.

### 3.3 Sonochemiluminescence (SCL) study of cavitation

**Fig. 6:**
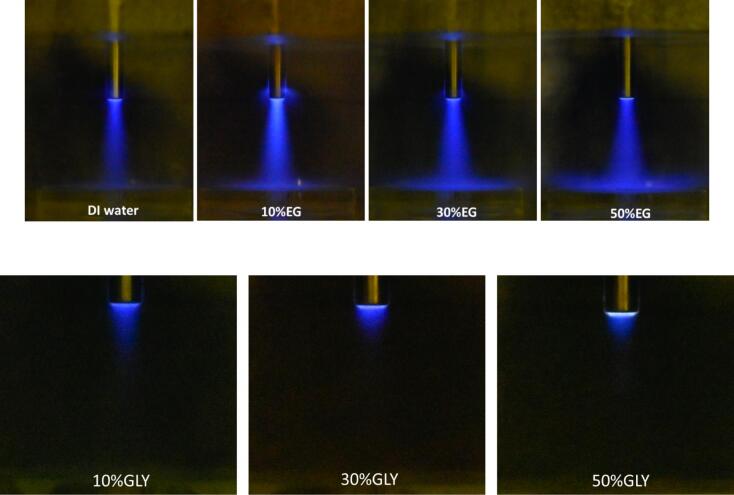
Sonochemiluminescence images of cavitation streaming under the 6 mm-Φ sonotrode operating at a power density of 354 W/cm^2^, in solutions of deionised water containing different vol% of ethylene glycol (top) or glycerol (bottom).

The SCL images depicted in Fig. 6 also reveal cavitation streaming beneath the sonotrode. The intensity and volume of the luminescence indicate a correlation with the swPCD analyses for solutions containing different proportions of EG and Gly. Specifically, the addition of EG has been observed to increase cavitation activity. In each of the SCL images, the highest cavitation density is observed in clusters on or near the sonotrode front surface, with intensity gradually decreasing along the narrow stream distally, in line with the acoustic streaming observed in the high-speed imaging of Fig. 3. The solution with 10 % EG appears to exhibit the highest density of bubbles, decreasing with further addition of EG. Furthermore, the acoustic streaming of a greater density of bubbles away from the sonotrode tip is apparent for the solution’s incorporation EG. It should be noted that SCL images primarily depict the activity of the cavitating bubbles as a whole, rather than highlighting the shockwaves generated by the cavitation cluster. We hypothesise that this is due to the shockwaves generated by the cavitation cluster having insufficient strength, in comparison to the individual bubble collapses, to create OH^•^ radicals.

According to the V_RMS_ values given in Fig. 5 it would be expected that the delamination efficiency would increase with any amount of EG content, up to 30 vol%, if the shockwaves alone were the most important aspect of the delamination. If cavitation intensity was the controlling factor, it would be expected that 10 vol% EG would have the best delamination because of the most intense cavitation observed at the sonotrode tip, as seen by the pale colour showing the greatest intensity of light. However, it would also reasonably be anticipated that the solutions with higher glycol contents should have a better delamination efficiency than water on its own.

In the SCL images of solutions containing 10, 30 and 50 vol% Gly, truncated cavitation streams are observed, with the greatest intensity of luminescence concentrated near the sonotrode tip. This might be due to the high viscosity of Gly solution which hinders the movement of cavity bubbles. There is also the possibility that Gly may dampen the SCL and hinder the generation of OH^•^ radicals.

### 3.4 Delamination of lithium-ion battery (LiB) cathode

**Fig. 7:**
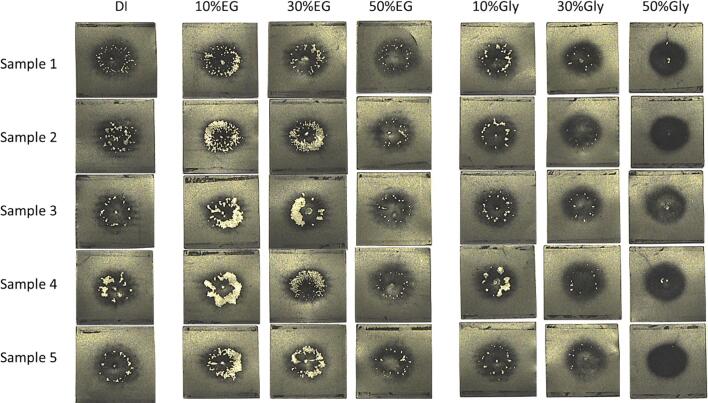
Comparison of ultrasonic delamination strength of different solutions on a 3 cm × 3 cm piece of LiB cathode. The delamination was performed using a 20 mm-Φ sonotrode for 10 s, at a sample-to-sonotrode distance of 5 mm, operating at an intensity of 119 W/cm^2^.

When the delamination behaviour of the LiB cathode sheet is considered in the context of the impact of glycol content on acoustic pressure, the results become clearer. The results of the cathode delamination are shown in Fig. 7. Initially, the cathode coating appears black. After the sonication, the cathode coating peels away to expose the bright aluminium foil beneath. The magnitude of the V_RMS_ values (observed in Fig. 5) for the solutions containing different vol% of EG correspond directly to the extent of delamination due to the peeling action of the shockwaves. In the more viscous systems containing Gly, where shockwaves are dampened and peeling action is diminished, the intensity of the dark bombardment mark correlates directly to the increase in focused microjets along the sonotrode axis line.

**Fig. 8:**
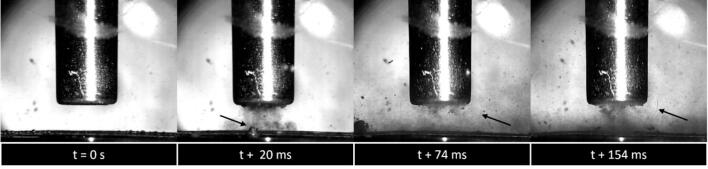
Representative frames from a high-speed imaging sequence of LiB delamination in a 10 vol% EG solution evidencing bubble jetting on the surface of the LiB (arrowed at 20 ms) and shockwaves emitted from the bubble cluster at the distal end of the sonotrode (arrowed at 74 and 154 ms).

Delamination is observed to occur through a combination of cavitation-mediated phenomena, particularly shockwaves and micro-jets on or near the surface (Fig. 8), and as is observed in other delamination studies [11]. The shockwaves act to peel off the coating layer, whereas the micro-jet impacts create pits and nucleation sites on the coating surface. For all samples, the resulting round impact site is clearly visible after the 10 s of sonication. Note that in the previously reported data [19], complete removal of the active material was achieved by moving the electrode material under the sonotrode. Crack propagation is thought to be an important mechanism for removal of the active coating layer.

The addition of 10 to 30 vol% EG significantly increased the delamination rate of the cathode active material coating when compared to deionised water alone, indicating strong shockwaves in these solutions. However, when the EG content was increased to 50 vol%, the delamination rate decreased to a level similar to deionised water. The cathode electrode coating with PVdF binder is elastic and difficult to fully delaminate through localised jetting alone, which only chips away small pieces of the coating. However, the additional contribution from intense bubble collapse shockwaves assists in the delamination process, leading to the formation of cracks following fatigue, allowing large flakes of active material coating to become separated from the surface.

The addition of Gly in all vol% caused a decrease in delamination rate compared to DI water, indicating a decrease in shockwave strength. However, the round pitting became darker, indicating increased jetting activity. This can be attributed to changes in viscosity and surface tension in the water-Gly solution. Gly has a higher viscosity than EG (supplementary material Table 1S), and excessive viscosity can dampen and attenuate shockwaves, resulting in reduced propagation distance from the sonotrode. However, cavitating bubbles may accumulate on the sample surface, due to delayed cavity bursts, so as to increase the population of jetting bubbles within the liquid volume (cavitation activity) on the sample surface.

### 3.5 Erosive force of cavitation

**Fig. 9:**
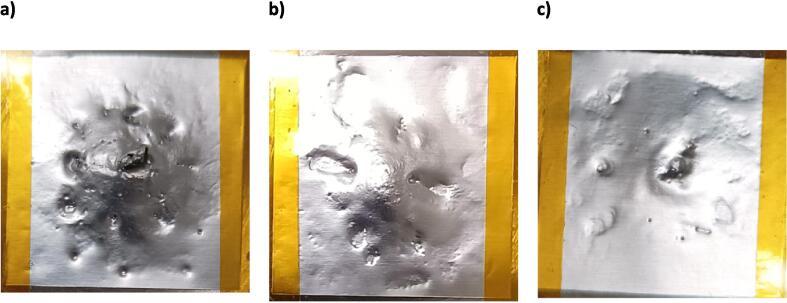
Cavitation erosion and shockwave action on a 3 cm × 3 cm piece of aluminium foil (30 μm thick) after a 3 s sonication, at a distance of a) 2.5 mm, b) 5.0 mm, and c) 10.0 mm. The sonotrode diameter is 20 mm, operating at a power density of 119 W/cm^2^, and the solvent was deionised water with 10 v/v% EG. The yellow/orange material at the top and bottom of the images is the Kapton tape used to attach the sample to the holder.

One issue in the recovery of cathode and anode active materials from LiB battery electrodes is that of purity. During the application of ultrasound, the shockwaves flex the electrode material multiple times to induce stress fracturing of the coating, and cavitation micro jets help to cause erosion. The high energies involved in the delamination process can cause crumpling, buckling, and pitting of the foil current collectors [19]. To better understand the damaging effect of the cavitation on a sample surface, the effect of distance from the sonotrode was tested on thin (30 μm thick) aluminium foil, with the resulting damage illustrated in Fig. 9. The corrugated deformation, characterised by wrinkles to the foil and small pitting on the surface, is attributed to the bubble-collapse phenomena discussed previously. In particular, the shockwaves from the cavitation cluster, whose strength diminishes rapidly with distance from the sonotrode tip and direct impact of bubble jets from non-spherically collapsing bubbles at the surface. At a distance of 2.5 mm, the corrugation indentation is nominally circular, with a diameter of around 15 mm. Pits of varying diameters (ca. 1–100 μm) are created by high-speed micro jets and associated shockwaves from individual cavitation bubbles on or near the surface. Some tearing of the aluminium foil is observed. At a distance of 5.0 mm, the region of deformation is wider. At 10 mm distance, there is only a mild deformation of the aluminium foil due to the cavitation, and very few pits, suggesting that it would be unlikely that appreciable delamination of any coated materials will occur at greater distances from the sonotrode. The rapid decrease in acoustic strength (both contributed by the major shockwaves and direct surface jets) is attributed to the attenuation of sound in the liquid medium and the shielding effect of the cavitation cluster. In this regard, the high-density bubble layer on the distal surface of the sonotrode serves as a buffer to absorb and scatter the ultrasound waves (vibration energy).

For a rigid aluminium plate (0.5 mm thick), the resulting deformation is depicted in Figure S1. Unlike the thin aluminium foil, long-range shockwaves did not induce any corrugated deformation on the surface. Instead, more visible deformation is observed in the form of micron-scale dents created by cavity micro jets on or near the surface. In the presence of 10 vol% EG, the erosion of the aluminium plate is stronger and more evenly spread across the area than that in deionised water. Reduced erosion is observed for the sample containing 30 vol% EG, suggesting that the erosive action of micro jets is higher than that of shockwaves from the cavitation cluster in the higher-viscosity solution.

### 3.6 Summary

Delamination efficiency of the LiB cathode sheet in aqueous media was seen to vary as a function of glycol type and content. The addition of EG resulted in improved delamination relative to deionised water alone, up to an EG content of 30 vol%, whereas the addition of Gly decreased delamination efficiency. The effect of different vol% of ethylene glycol and glycerol on ultrasonic cavitation in aqueous systems was investigated using a swPCD to detect shockwave content within the cavitation emissions. This was coupled with high-speed imaging to observe the development of the cavitation and observe the shockwaves generated, and SCL observations to visualise the active cavitation regions at the tip of the sonotrode. The addition of 10 to 30 vol% ethylene glycol results in increased shockwave content, by measure of *V_RMS_*, due to the increase in solvent viscosity and lower surface tension. Above 30 vol%, the viscosity of the solution becomes high enough to cause an acoustic dampening effect. This behaviour was observed whether the swPCD was placed beside or underneath the sonotrode tip. The presence of glycerol in the solution has minimal effect on *V_RMS_* when the swPCD was placed beside the sonotrode, but a consistent increase in *V_RMS_* was observed with glycerol content when the swPCD was placed directly underneath. These observations were indicative of the higher viscosity of the solutions containing glycerol causing the attenuation of acoustic pressure away from the sonotrode axial line and focussing of the acoustic pressure in a narrow column along the sonotrode axis line.

SCL observations of solutions containing different vol% of ethylene glycol confirmed that the highest density of cavitating bubbles is observed in clusters on or near the sonotrode front surface, with a gradual decrease in intensity along the axial line. The highest density of bubbles at the sonotrode tip was observed for an ethylene glycol content of 10 vol%, which decreased upon further addition of ethylene glycol, due to delayed burst of cavitation bubbles in a solution with increased viscosity.

## 4 Conclusions

Our investigation reveals that the active force behind the delamination of a coated film, such as a LiB cathode electrode, stems from two different sources: the violent shockwaves emitted from cavitation clusters developed in proximity to the sonotrode tip, and the individual jetting bubbles located on or near the sample surface. Shockwaves induce cracking and peeling of the coating, while cavity jets cause short-range dents and chips on the sample surface. The addition of EG to deionised water improves delamination efficiency, but there should be no more than 10–30 vol% present, otherwise the elevated viscosity causes significant dampening effects on the shockwaves. Gly is not a suitable additive to be used for the ultrasonic delamination of lithium-ion battery cathode materials, leading to the recommendation that only the lower viscosity glycols should be used as additives. The swPCD cavitation detector is proven to be a practical and effective device for analysing an unknown solution's cavitation properties, and hence delamination strength, in comparison to water. This permits selection of the solution with the highest delamination strength for a film delamination at fast speeds.

These observations will be critically important for any scaled-up battery recycling processes that intend to employ ultrasound as a means of active material delamination from the current collector foils. This work could also be expanded into the selection of appropriate solvent blends for the delamination of other composite materials that have a solid substrate and a brittle coating, such as the removal of metal oxide active materials from fuel cell catalyst-coated membranes, or for the pulverisation of semiconductor legs from thermoelectric devices, or even exfoliation of graphene sheets from graphite.

## CRediT authorship contribution statement

**Chunhong Lei:** Writing – review & editing, Writing – original draft, Investigation, Conceptualization. **Ben Jacobson:** Writing – review & editing, Software, Investigation. **Jennifer M. Hartley:** Writing – review & editing, Investigation. **Sean Scott:** Writing – review & editing, Investigation. **Iwan Sumarlan:** Writing – review & editing, Investigation. **Andrew Feeney:** Writing – review & editing, Supervision, Project administration, Funding acquisition. **Paul Prentice:** Writing – review & editing, Supervision, Project administration, Funding acquisition. **Karl S. Ryder:** Writing – review & editing, Supervision, Project administration, Funding acquisition. **Andrew P. Abbott:** Writing – review & editing, Supervision, Project administration, Funding acquisition, Conceptualization.

## Declaration of competing interest

The authors declare that they have no known competing financial interests or personal relationships that could have appeared to influence the work reported in this paper.
