# Supplementary material for: Effect of organic solvent additives on the enhancement of ultrasonic cavitation effects in water for lithium-ion battery electrode delamination
Source: Ultrason Sonochem. 2024 Aug 31;110:107049. doi: 10.1016/j.ultsonch.2024.107049 (PMC11403513; doi:10.1016/j.ultsonch.2024.107049)
Supplement: Supplementary Data 1 [file mmc1.docx]

**Effect of organic solvent additives on the enhancement of ultrasonic strength in water for lithium-ion battery electrode delamination**

*Chunhong Lei ^a^*, Ben Jacobson ^b^, Jennifer M. Hartley ^a^, Sean Scott ^a^, Iwan Sumarlan ^a,c^, Andrew Feeney ^b^, Paul Prentice ^b^, Karl S. Ryder ^a^, Andrew P. Abbott ^a^*

*^a^ School of Chemistry, University of Leicester, Leicester, LE1 7RH, UK*

*^b^ James Watt School of Engineering, University of Glasgow, Glasgow, G12 8QQ, UK*

*^c^ Department of Chemistry, University of Mataram, Lombok, Indonesia.*

* cl415@leicester.ac.uk

**Supplementary Information**

| **Table S1.** Thermophysical properties of water, ethylene glycol, and glycerol in air at 25 ^o^C.^1^ | | | | |
| --- | --- | --- | --- | --- |
| **Solvent** | **Density**  **(g/mL)** | **Vapour pressure (mmHg)** | **Viscosity**  **(cP)** | **Surface tension (N/m)** |
| Water | 1.0 | 23.8 | 0.890 | 0.072 |
| EG | 1.11 | 0.089 | 16.1 | 0.048 |
| Gly | 1.26 | 0.0002 | 954 | 0.064 |

| 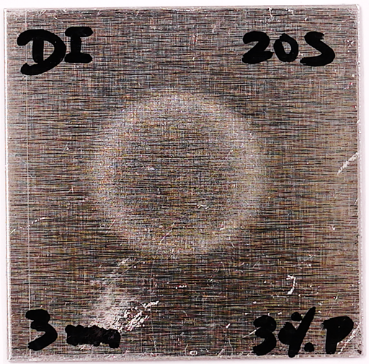 | 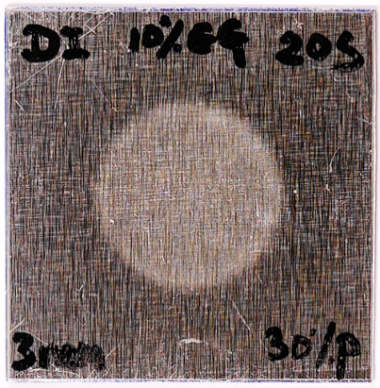 | 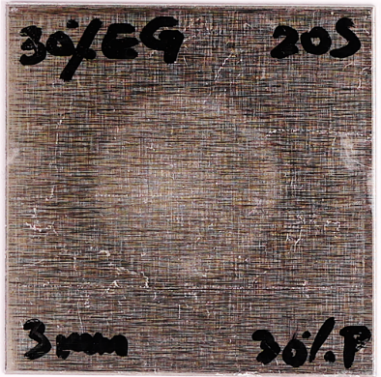 |
| --- | --- | --- |
| **Figure S1.** Cavitation erosion on a 3 cm x 3 cm rigid aluminium plate (0.5 mm thick) after the application of 20 s of ultrasound, at a distance of 3 mm from the sonotrode, in a) deionised water, b) deionised water + 10 vol% EG, and c) deionised water + 30 vol% EG. The sonotrode diameter is 20 mm, operating at a power density of 119 W/cm^2^. | | |

| **a) Deionised water** | **b) 10 vol% EG** |
| --- | --- |
| 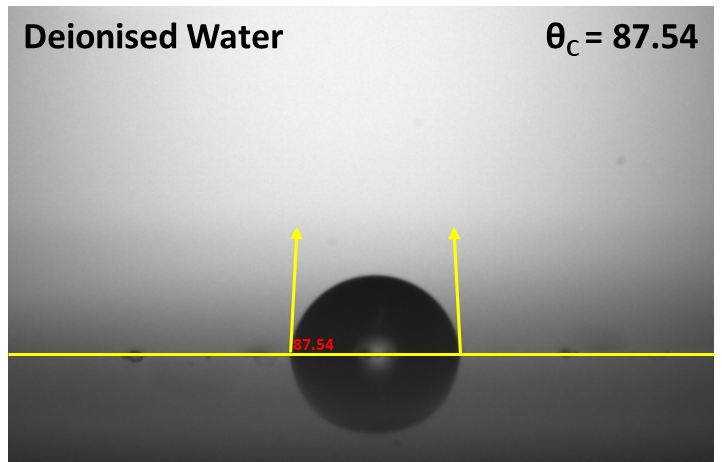 | 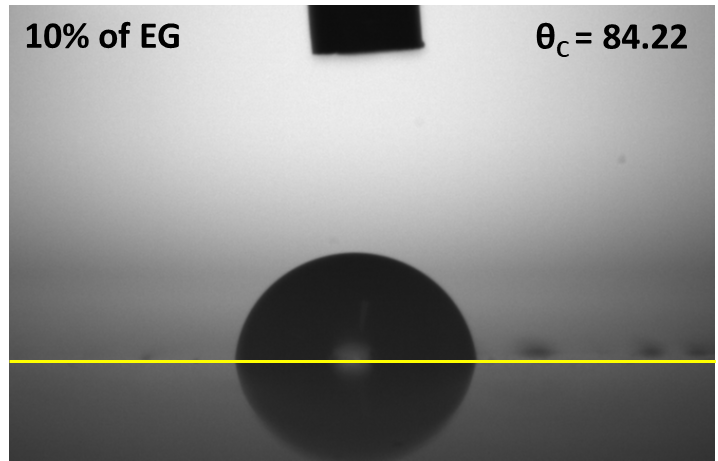 |
| **c) 20 vol% EG** | **d) 30 vol% EG** |
| 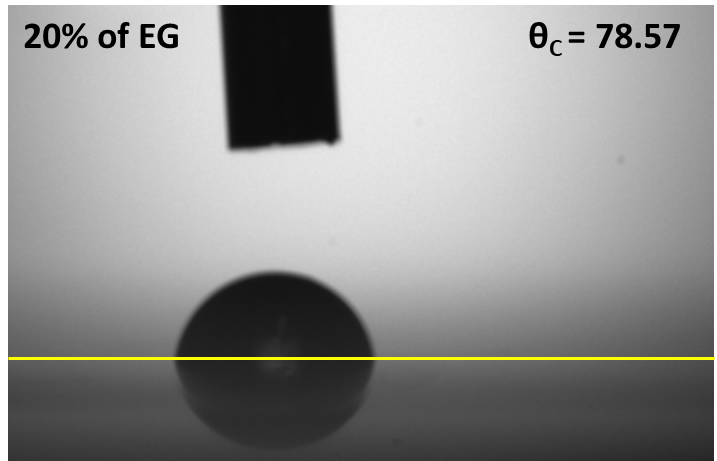 | 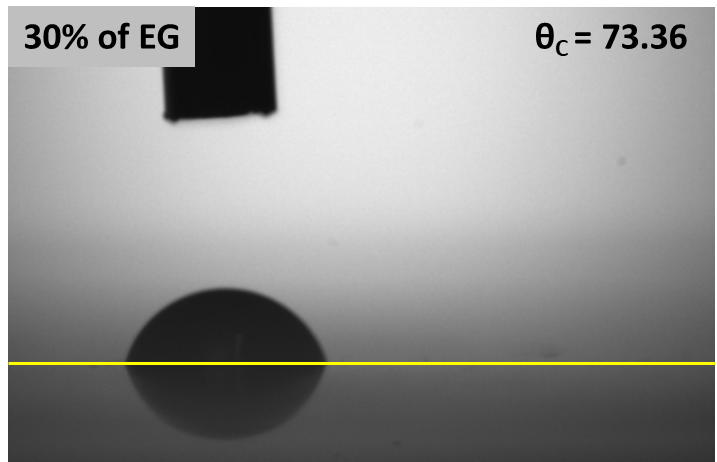 |
| **e) 40 vol% EG** | **f) 50 vol% EG** |
| 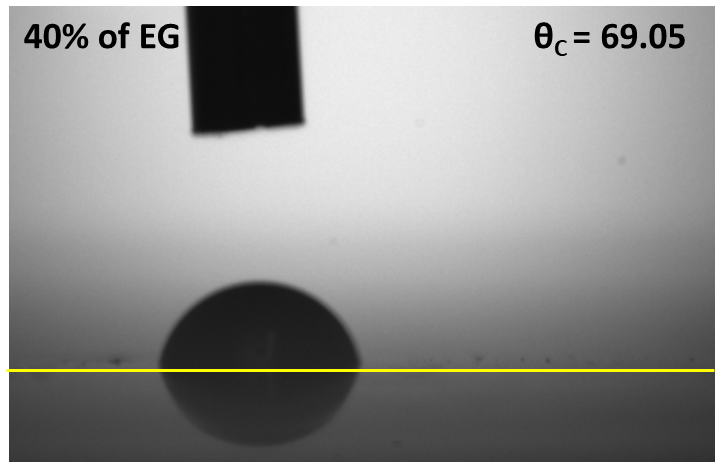 | 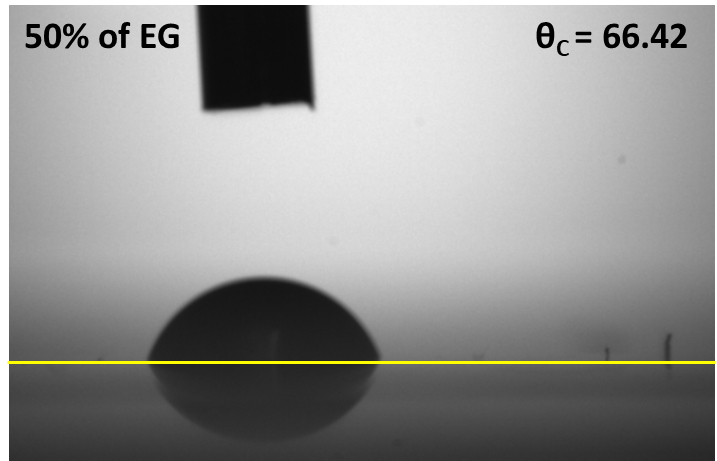 |
| **Figure S2.** Contact angle images for different solutions of ethylene glycol (EG) in water: a) deionised water, b) 10 vol% EG, c), 20 vol% EG, d) 30 vol% EG, e) 40 vol% EG, and f) 50 vol% EG. The substrate was a PVDF film cast onto a glass microscope slide. Note that the black angular shape at the top of the image is the tip of the syringe used to apply the sample, and the yellow line is drawn on to the image to guide the eye as to where the surface of the substrate is. | |

| **a) 10 vol% Gly** | **b) 20 vol% Gly** |
| --- | --- |
| 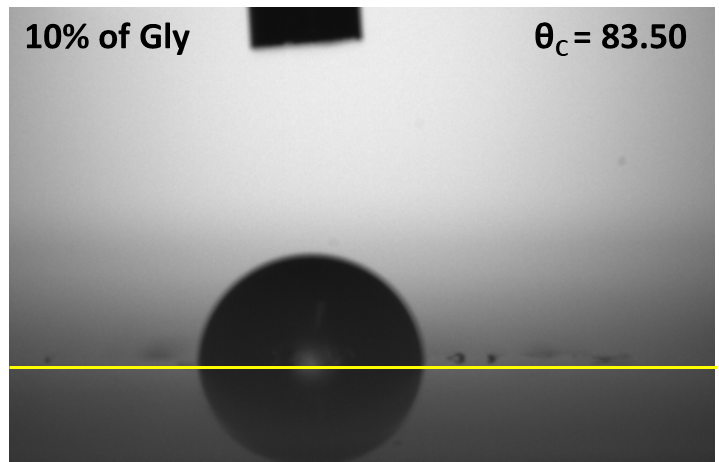 | 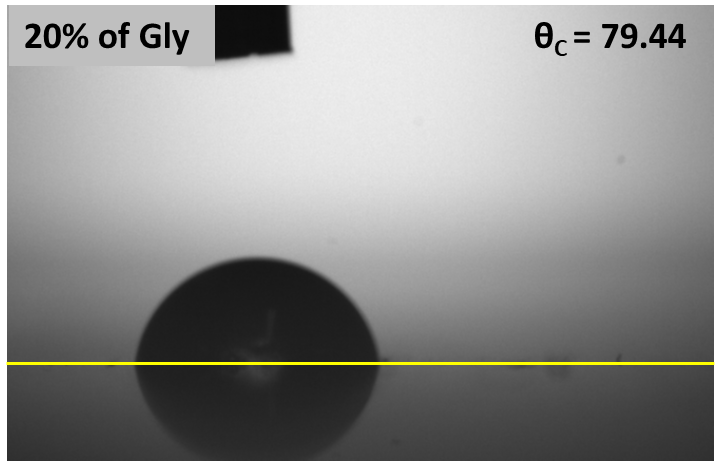 |
| **c) 30 vol% Gly** | **d) 40 vol% Gly** |
| 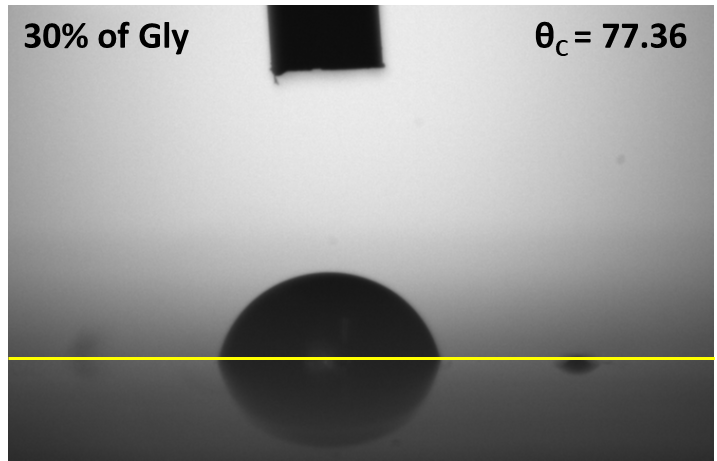 | 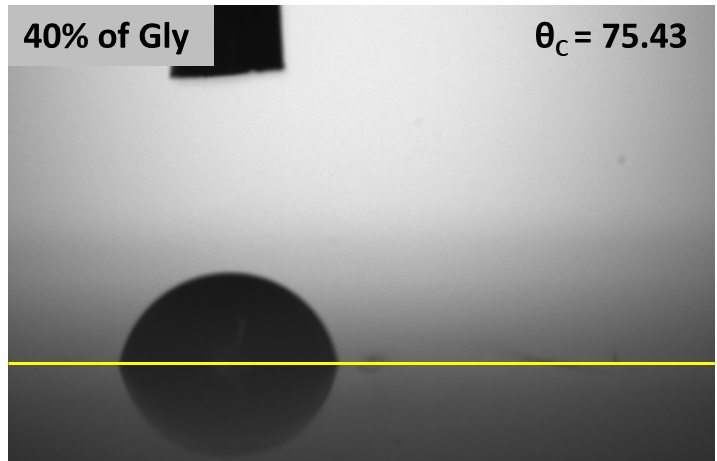 |
| **e) 50 vol% Gly** |  |
| 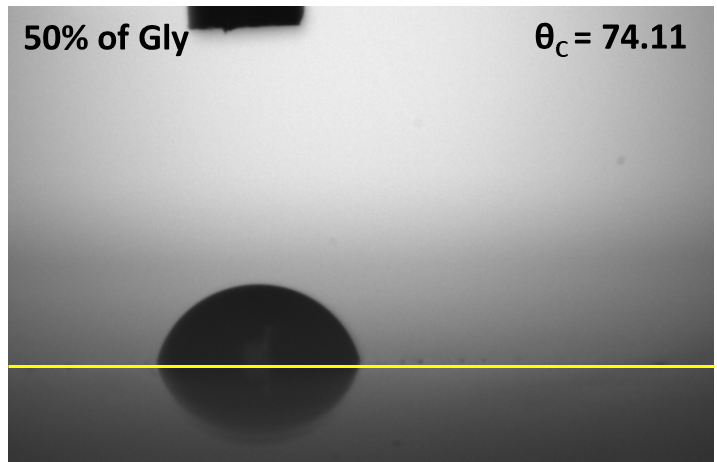 |  |
| **Figure S3.** Contact angle images for different solutions of glycerol (Gly) in water: a) 10 vol% Gly, b), 20 vol% Gly, c) 30 vol% Gly, d) 40 vol% Gly, and e) 50 vol% Gly. The substrate was a PVDF film cast onto a glass microscope slide. Note that the black angular shape at the top of the image is the tip of the syringe used to apply the sample, and the yellow line is drawn on to the image to guide the eye as to where the surface of the substrate is. | |

**References**

1. *CRC Handbook of Chemistry and Physics, 87th ed*, CRC Press/Taylor and Francis Group, 2006.
